# Transamniotic Fetal Delivery of Cystic Fibrosis (CF) Transmembrane Conductance Regulator (CFTR) mRNA

**DOI:** 10.1096/fba.2025-00288

**Published:** 2026-04-08

**Authors:** Kamila Moskowitzova, Ashlyn E. Whitlock, Yash V. Shroff, David Zurakowski, Dario O. Fauza

**Affiliations:** ^1^ Department of Surgery Boston Children's Hospital/Harvard Medical School Boston Massachusetts USA

**Keywords:** cystic fibrosis, fetal mRNA therapy, TRANAT, transamniotic fetal therapy, transamniotic mRNA therapy, transmembrane conductance regulator

## Abstract

We sought to determine whether a cystic fibrosis transmembrane conductance regulator (CFTR) mRNA could be administered to the fetus via the transamniotic route as a potential strategy for the perinatal management of cystic fibrosis‐associated meconium ileus. Nine pregnant Sprague Dawley dams underwent volume‐matched intra‐amniotic injections in all their fetuses (*n* = 109) of either a suspension of a human CFTR mRNA encapsulated by a semi‐synthetic composite, lipopolyplex (mRNA group; *n* = 98), or of a suspension of the same composite free of mRNA (control group; *n* = 11), on gestational day 17 (E17; term = E21). At daily time points until term, fetal small bowel and lungs, along with maternal serum, were quantitatively screened for the human CFTR protein by ELISA. Statistical analysis was by the nonparametric Wilcoxon rank sum test with Bonferroni adjustment. Overall fetal survival was 85.3% (93/109), with no significant differences between the groups or time points. When controlled by mRNA‐free injections, human CFTR was detected in the small bowel from the mRNA group at all time points (*p* < 0.001 for all), increasing initially over time. Human CFTR was detected comparably in the lungs from both groups, suggesting interspecies homology at that anatomical site. No human CFTR was detected in maternal serum. Encapsulated exogenous mRNA encoding for the cystic fibrosis transmembrane conductance regulator protein can be incorporated and translated by fetal small bowel cells after simple intra‐amniotic injection in a healthy rodent model. Transamniotic mRNA delivery could become a novel strategy for the perinatal management of meconium ileus associated with cystic fibrosis.

## Graphical abstract

The transamniotic route has been shown to be a practical and effective method for delivering exogenous mRNA to the fetus by hematogenous routing directly through the amnio‐placental interface. This study demonstrates that exogenous encapsulated mRNA encoding for human cystic fibrosis transmembraneconductance regulator (CFTR) can be incorporated and translated by fetal cells following simple intra‐amniotic injection in a healthy rat model. Transamniotic mRNA delivery could become a novel strategy for the prevention or management of perinatal complications of cystic fibrosis.

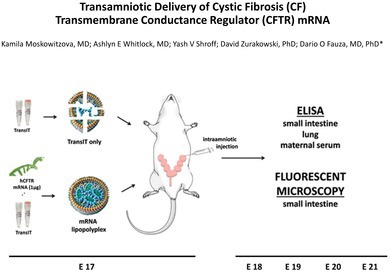

## 1 Introduction

As a monogenic autosomal recessive disease, cystic fibrosis (CF) is a natural candidate for novel therapies based on exogenous mRNA administration. Indeed, clinical trials of mRNA‐based therapies as inhalation products for respiratory complications of CF are already a reality [1, 2]. A central component of CF pathophysiology is a mutation in the CF transmembrane conductance regulator (CFTR), a cell membrane protein serving as a chloride/anion channel in numerous cell phenotypes, leading to CFTR absence or malfunction. The gastrointestinal tract is commonly affected, often as early as the perinatal period, so that newborns with CF may suffer from meconium ileus as a result of that, followed by respiratory complications not long thereafter [3, 4, 5]. The diagnosis of CF can be made early in pregnancy via genetic screening, and certain fetal ultrasound findings may be predictive of meconium ileus after birth [6].

Exogenous encapsulated mRNAs have been successfully delivered to the fetus via simple intra‐amniotic injections, reaching multiple anatomical sites, including the intestines and lungs [7, 8]. In this study, we sought to determine whether the transamniotic route could be a viable alternative for the delivery of CFTR mRNA to the fetal intestine and lung in a healthy rat model. If so, this could become a novel strategy for the perinatal management or prevention of meconium ileus in neonates with CF.

## 2 Materials and Methods

This study was approved by Boston Children's Hospital Institutional Animal Care and Use Committee under protocol 20‐03‐4152.

### 2.1 mRNA Formulation and Encapsulation

A human CFTR (hCFTR) mRNA sequence was obtained commercially (NCBI: NM_000492; Ribo Pro, The Netherlands) under a custom order including the following modifications: Cap1 to deimmunize the sequence, 150 nt PolyA‐tail, and proprietary sequence optimization to yield increased translation into the final protein product. Just prior to injection in vivo, the custom hCFTR mRNA was encapsulated into semi‐synthetic lipolyplex particles made of a self‐assembling composite lipid and cationic polymer (TransIT; Mirus Bio, Madison, WI) consisting of a reagent proper and an mRNA boost reagent, using the TransIT‐mRNA transfection kit (Mirus Bio) in accordance with the manufacturer's instructions. Briefly, 1 μg of mRNA (1 μg/μL stock) was suspended in 45 μL of phosphate‐buffered saline (PBS), then 2 μL of each of the kit's two components (reagent and boost reagent) were added, followed by 5 min incubation at room temperature to allow for encapsulation. Intra‐amniotic injections were performed as described below.

To evaluate the efficiency of mRNA encapsulation into the lipopolyplexes, the Quant‐iT RiboGreen assay (Thermo Fisher Scientific, Waltham, MA) was utilized as previously described [9]. Briefly, known amounts of encapsulated mRNA into lipopolyplex (1 μg/50 μL stock) were mixed with fluorescent RiboGreen reagent (Thermo Fisher Scientific). The amount of free‐floating mRNA in that solution was then measured on a microplate reader (BMG Labtech, Cary, NC) by fluorescent detection at 490 nm and 520 nm excitation and emission, respectively. Encapsulation efficiency was determined by subtracting the amount of free mRNA in the lipopolyplex solution (lipopolyplex mRNA) from the total mRNA originally used (naked mRNA) and expressed as a percentage. The same procedure was performed in parallel using naked mRNA in PBS (1 μg/50 μL stock) instead of encapsulated mRNA, as positive controls.

### 2.2 Intra‐Amniotic Injections

**FIGURE 1:**
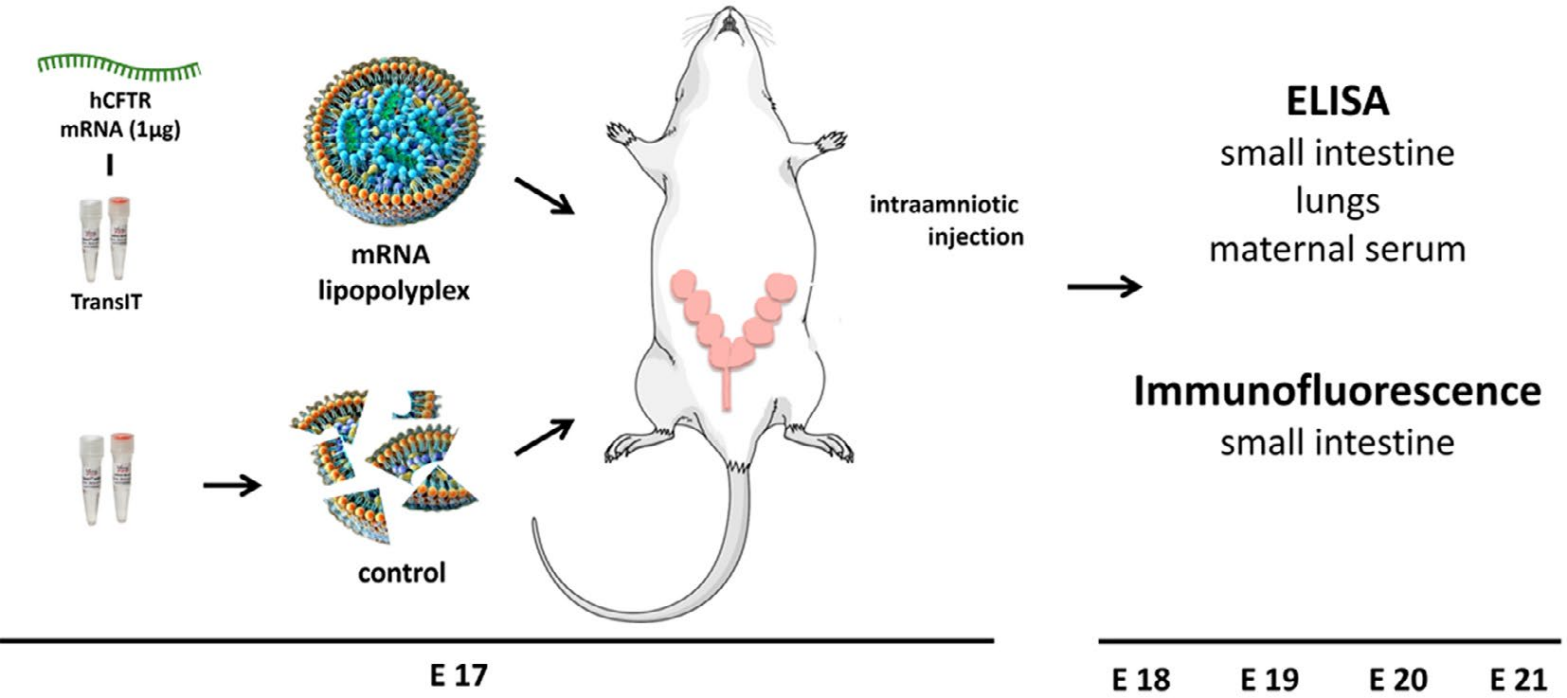
Overview of the experimental protocol. E = gestational day (term = E21).

The overall experimental design is shown in Figure 1. Nine time‐dated pregnant Sprague Dawley dams (Charles River Laboratories, Wilmington, MA) underwent surgery for direct intra‐amniotic injections on gestational day 17 (E17, term = E21–22) as previously described [10]. In brief, dams were sedated with 2%–4% inhaled isoflurane (Patterson Veterinary, Greeley, CO) in 100% oxygen. Following a midline laparotomy to expose the bicornuate uterus, all their fetuses (*n* = 109) received volume‐matched intra‐amniotic injections (50 μL) of either a suspension of a hCFTR mRNA lipopolyplex (mRNA group; *n* = 98) or a suspension of the encapsulation composite but free of mRNA (control group; *n* = 11) using a 33G non‐coring needle on a 100 μL syringe (both from Hamilton Company, Reno, NV). Injections were performed under direct vision with care to avoid injury to the fetus, placenta, or umbilical cord. The incision was closed in two layers, and powdered metronidazole (Unichem Pharmaceuticals, Hasbrouck Heights, NJ) was applied to the wound. Post‐operative analgesia was provided by extended‐release buprenorphine (Fidelis Pharmaceuticals, North Brunswick, NJ).

### 2.3 Specimen Procurement

Dams were euthanized by CO_2_ chamber at daily time points following the intra‐amniotic injections, from E18 to E21 (term) for the mRNA group and at E21 only for the control group. Samples from the fetal small intestine and lung were procured as previously described [11]. Small intestine samples from E19, E20, and E21 fetuses were divided into 2 halves, with one being immediately frozen for enzyme‐linked immunosorbent assay (ELISA) analyses, while the other was fixed in 10% formalin for histology. Intestinal samples from E18 animals were not divided due to their very small size and were only frozen for ELISA. Maternal blood was obtained via direct intracardiac puncture and centrifuged at 2000 rcf for 8 min for serum separation and storage. All sample freezing was by rapid dry ice‐ethanol baths followed by storage at −80°C until further processing.

### 2.4 hCFTR ELISA

Fetal small intestine and lung samples were standardized by weight and homogenized. Maternal serum samples were standardized by volume. All samples were screened for the presence of the hCFTR protein using a commercially available ELISA kit (Biorbyt, Cambridge, United Kingdom) in accordance with the manufacturer's instructions. The final hCFTR protein concentration in ng/mL was calculated after all samples and standards were run in duplicate and averaged.

### 2.5 Immunofluorescence

Immunofluorescent staining for hCFTR was performed on subsets of formalin‐fixed fetal small intestines to assess the topography of cellular distribution of the protein within the enterocytes. Three samples from the mRNA group, each from a different time point (E19–E21), and two samples from the control group at E21 were randomly selected. Samples were paraffin‐embedded and sectioned. Paraffin section slides were then deparaffinized and rehydrated following 15 min in xylene, twice. Specifically, 5, 5, and 5 min in 100%, 100%, and 75% ethanol, respectively, and 5 min in PBS at room temperature, repeated three times. Slides were heated up to 110°C for 15 min, then cooled down to room temperature for 30 min and washed with Tris‐buffered saline/Tris‐buffered saline with 0.1% Tween 20 detergent (TBS/TBST) for 5 min, three times. Slides were then blocked with normal goat serum and incubated with primary antibodies (hCFTR C‐terminus antibody; Biotechne R&D Systems, Minneapolis, MN) overnight at 4°C. Following repeated TBS/TBST wash for 5 min three times, slides were incubated with secondary antibodies (Goat anti‐Mouse IgG, Alexa Fluor; Thermo Fisher Scientific) for 1 h at room temperature. Finally, slides were stained with nuclear 4′,6‐diamidino‐2‐phenylindole (DAPI). Immunolabeled sections were examined on a Zeiss LSM 880 Confocal Microscope (Zeiss, Oberkochen, Germany) under oil lenses. Representative images were taken at 63× magnification.

### 2.6 Statistical Analyses

Fetal survival comparisons between the groups were made using Fisher's exact test. ELISA data were compared by a nonparametric Wilcoxon rank sum test. Statistical significance was defined by a Bonferroni‐adjusted *p* < 0.05.

## 3 Results

The encapsulation rate of the hCFTR mRNA into the lipopolyplex was 99.5%. Overall fetal survival was 85.3% (93/109), with no significant differences between the groups or time‐points (*p* > 0.067 for all). There was no maternal mortality.

### 3.1 Fetal hCFTR Production

**FIGURE 2:**
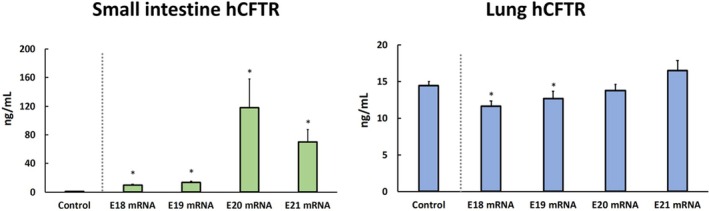
Human CFTR (hCFTR) protein levels by ELISA in fetal rats at E18–E21 for the mRNA group and at E21 only for the control group in (A) small intestine and (B) lungs. Data presented as mean ± SEM, *p < 0.05 vs. control.

When controlled by mRNA‐free injections, hCFTR protein was detected in the fetal small intestine of the mRNA group at all time points (*p* < 0.001 for all, Figure 2A). The amount of intestinal hCFTR tended to increase over three days after intra‐amniotic mRNA delivery (average at E20 = 118 ± 40 ng/mL), with persistently high levels at term (average at E21 = 70 ± 17 ng/mL). Interestingly, the hCFTR protein was detected in fetal lung in both the mRNA and control groups, with even significantly decreased amounts at E18–19 in the mRNA group vs. control (*p* < 0.012 for both; Figure 2B), suggesting the possibility of a significant degree of CFTR interspecies homology, particularly at this anatomical site. When controlled by mRNA‐free injections, no hCFTR was detected in any maternal serum sample.

**FIGURE 3:**
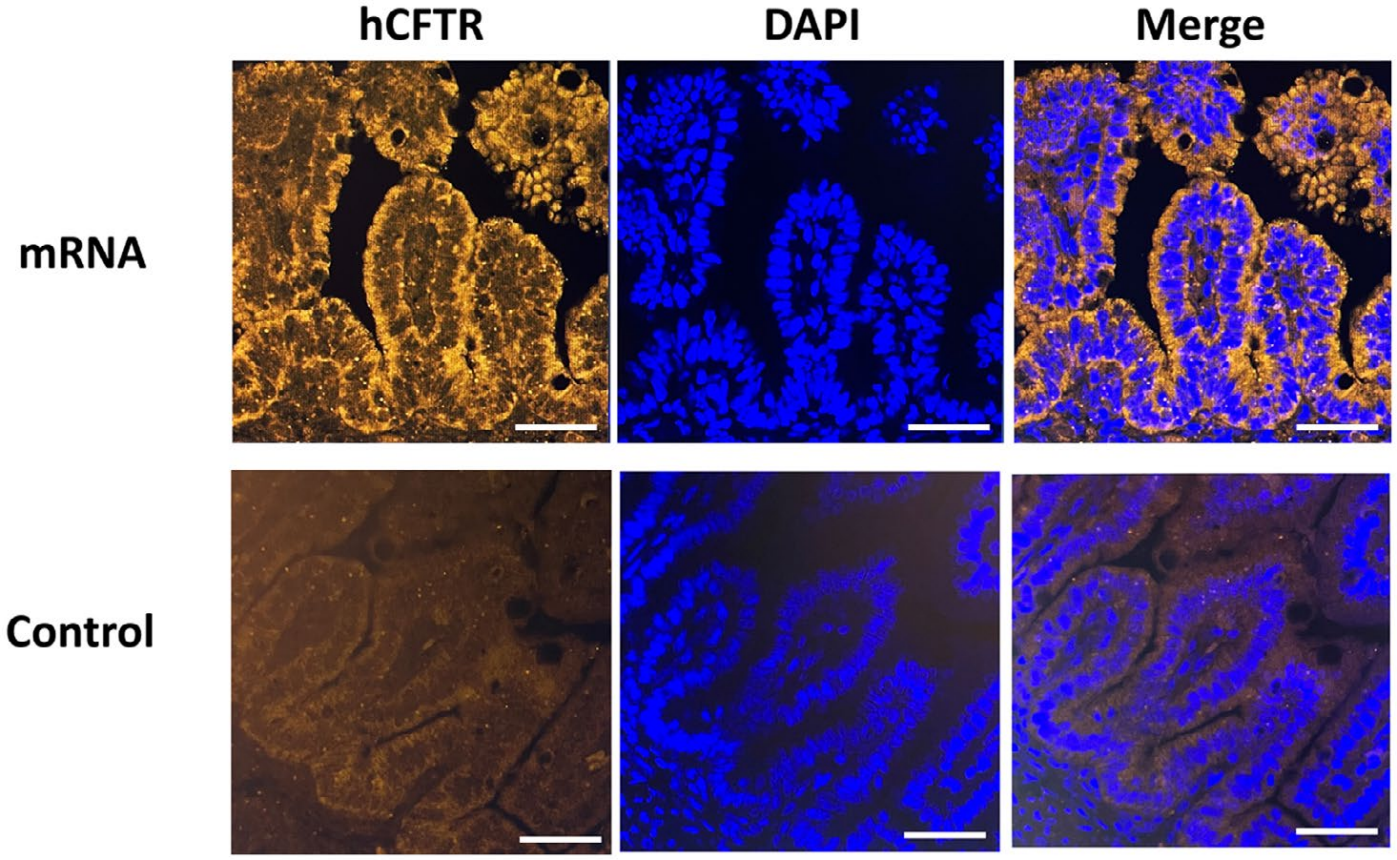
Representative images of fluorescence microscopy of fetal small intestine from the mRNA group (at E20) and the control group (at E21). Tissue sections were stained for hCFTR (anti‐hCFTR, Cy3; orange) and nuclei (DAPI, blue). Magnification: 63X. Scale bar: 40 μm.

Representative immunofluorescent images showed a strong, concentrated fluorescent signal at the apical membranes of enterocytes in the small intestine in the mRNA group, in accordance with typical apical hCFTR localization (Figure 3). No hCFTR signal was observed in the control group.

## 4 Discussion

Perhaps not surprisingly, it was initially thought that mRNA administered into the amniotic fluid would have the same fate as virtually anything delivered to that medium,i.e., it would be only swallowed and/or aspirated by the fetus [8]. A recent study has shown that, while this certainly happens, mRNA injected in the amniotic fluid can actually also undergo hematogenous routing through the gestational membranes and placenta and from there reach multiple fetal anatomical sites [7]. Although this may also possibly apply to transamniotic CFTR mRNA delivery, it was beyond the scope of this study to perform a comprehensive pharmacokinetics analysis of that particular mRNA “product”. Given the role of CFTR absence or malfunction in neonatal meconium ileus associated with CF, as well as in the respiratory complications that may ensue not long after birth, a focus on the intestines and lungs was justifiable in a first experiment such as this.

Our data showed consistent and enduring cellular incorporation of the hCFTR mRNA in the fetal intestine and its local translation to the hCFTR protein following transamniotic delivery. Given the minimally invasive nature of an amniocentesis and the fact that the fetus is frequently swallowing amniotic fluid, it is reasonable to speculate that this form of fetal mRNA delivery may offer advantages over an eventual postnatal form of administration, which has yet to be proven viable for the intestine. It has been shown, however, that encapsulated hCFTR mRNA can be incorporated by and function in adult mice's lungs for 6–14 days following intravenous or intratracheal nebulized administration [12, 13]. Even if postnatal administration proves viable, an additional benefit of the fetal delivery is the presence of the protein already at birth, which conceivably could be more effective in preventing meconium ileus in that patient population.

Clinically relevant CFTR mutation patterns and their consequences range from transcription disruptions leading to misfolded CFTR not reaching apical cell membranes to properly positioned chloride channel/CFTR proteins that are severely malfunctioning so as to not allow for adequate ion transport [14]. Our fluorescence microscopy data showed that a majority of the hCFTR protein was localized in the expected normal apical topography within the enterocytes. This is an encouraging finding, to be considered in the light of the fact that it has been previously shown in a transgenic porcine model of CF‐associated meconium ileus that as little as 20% of CFTR mRNA function in the intestine is sufficient to prevent meconium ileus [15].

It was intriguing to detect hCFTR protein in the fetal lungs from the control group at comparable or even higher levels to those detected in lungs from the mRNA group. One natural, albeit at this point speculative explanation could be that there is a degree of interspecies homology at this particular anatomical site, as in fact previously suggested by others [16, 17]. Another conceivable, though also speculative, explanation has to do with the mRNA product that we used. It was prepared via a proprietary sequence optimization technology developed by Ribo Pro that increases protein expression on average fivefold by removing toll‐like receptors that activate sub‐sequences that negatively impact protein expression. Notably, specimens from the control group were only procured at term. We cannot rule out the possibility that eventual comparisons matched by time point could have yielded different results. Besides these uncertainties, another limitation of this study is the fact that the functionality of the hCFTR protein could not be investigated. Yet, such an analysis could only be justified if the protein was present at all, preferably at its normal topography, hence this first experiment with such a focus.

Limitations notwithstanding, these data show that exogenous mRNA encoding for hCFTR protein can be incorporated and translated by fetal small intestine cells after simple intra‐amniotic injection in a healthy rat model. Transamniotic nucleic acid therapy (TRANAT) could become a novel strategy for the perinatal management and possibly prevention of meconium ileus associated with cystic fibrosis.

## Author Contributions

Conceptualization: K.M., D.O.F.; Data curation: K.M., A.E.W., Y.V.S.; Formal analysis: K.M., D.Z., D.O.F.; Funding acquisition: D.O.F.; Investigation: K.M., A.E.W., D.O.F.; Methodology: K.M., D.Z., D.O.F.; Project administration: K.M., D.O.F.; Resources: K.M., D.O.F.; Software: K.M., D.Z.; Supervision: D.O.F.; Validation: K.M., D.Z., D.O.F.; Visualization: K.M., D.O.F.; Writing – original draft: K.M., D.Z.; Writing – review and editing: K.M., D.O.F.

## Conflicts of Interest

The authors declare no conflicts of interest.

## Data Availability

Stored in repository.
